# Suture wear particles cause a significant inflammatory response in a murine synovial airpouch model

**DOI:** 10.1186/s13018-018-1026-4

**Published:** 2018-12-06

**Authors:** Vedran Lovric, Michael J. Goldberg, Philipp R. Heuberer, Rema A. Oliver, Dana Stone, Brenda Laky, Richard S. Page, William R. Walsh

**Affiliations:** 1 Surgical and Orthopaedic Research Laboratories, Prince of Wales Clinical School, University of New South Wales, Sydney, Australia; 2 Orthopaedic Department, St. Vincent Hospital, Vienna, Austria; 3 Barwon Centre for Orthopaedic Research and Education, School of Medicine, Deakin University, Waurn Ponds, Geelong, Victoria Australia

**Keywords:** Post-arthroscopic glenohumeral chondrolysis, Suture, Suture wear particles, Matrix metalloproteinase, Inflammatory response, Foreign body reaction

## Abstract

**Background:**

Commonly used contemporary orthopaedic sutures have been identified as a potential causative factor in the development of post-arthroscopic glenohumeral chondrolysis. Currently, little is known about the body’s immune response to these materials. The aim of this study was to examine the biological response of synovial tissue to three commonly used orthopaedic sutures, using a murine airpouch model.

**Methods:**

Fifty rats were used in this study (ten per group). An airpouch was created in each rat, and test materials were implanted. Test materials consisted of an intact polyethylene terephthalate suture with a polybutilate coating (suture A), an intact polyethylene suture braided around a central polydiaxannone core (suture B), an intact polyethylene/polyester cobraid suture with a silicone coating (suture C), and particles of suture C (particles C). Rats were sacrificed at 1 or 4 weeks following implantation. Histological (multinucleated giant cell count) and immunohistochemical (expression of matrix metalloproteinases MMP-1,-2,-3,-9,-13) markers of inflammation were examined.

**Results:**

Multinucleated giant cells were present in all specimens containing suture material but not in the control specimens. No significant differences were found in the number of giant cells between the intact suture groups at either time point. Significantly higher numbers of giant cells were noted in the particles C group compared to the intact suture C group at both time points (*p* = 0.021 at 1 week, *p* = 0.003 at 4 weeks). Quantitative analysis of immunohistochemical staining expression at 4 weeks showed that significantly more MMP (-1,-2,-9,-13) was expressed in the particles C group than the intact suture C group (*p* = 0.024, *p* = 0.009, *p* = 0.002, and *p* = 0.007 for MMP-1, MMP-2, MMP-9, and MMP-13, respectively). No significant difference was seen in the expression of MMP-3 (*p* = 0.058).

**Conclusions:**

There were no differences observed between the biological reactivity of commonly used intact orthopaedic sutures A, B, and C. However, wear particles of suture C elicited a significantly greater inflammatory response than intact suture alone. This was confirmed by increased numbers of multinucleated giant cells as well as MMP ( -1,-2,-9,-13) expression. Further studies are needed to determine whether this inflammatory response may play a role in the development of post-arthroscopic glenohumeral chondrolysis or interfere with biological healing. These findings have important clinical implications relating to surgical technique and surgical implant design.

## Background

Post-arthroscopic glenohumeral chondrolysis (PAGCL) is a rare, but significant complication of arthroscopic shoulder surgery. It refers to the death of glenohumeral chondrocytes, which causes rapid cartilage degeneration and extensive osteoarthritis, often in young patients. This produces devastating consequences for affected patients who experience significant pain and functional impairment, often necessitating salvage surgery. The aetiology of PAGCL is poorly understood; however numerous patient and surgical factors have been implicated [1]. Patient factors, such as genetics and type of glenohumeral pathology, may contribute towards an increased risk of developing PAGCL, with case reports suggesting that chondrolysis is most prevalent amongst young males with shoulder instability [2]. Furthermore, a number of surgical factors have been implicated in the pathogenesis of PAGCL, including intraarticular local anaesthetic, iatrogenic articular damage, suture anchors, infection, and the use of intraoperative radiofrequency devices. Some hypothesise that the high tensile suture materials used in arthroscopic surgery may also play a role; however, to date, there are no studies to support this assertion.

A number of different sutures are commonly used in arthroscopic shoulder surgery. Ethibond (Ethicon, Somerville, New Jersey, USA) is composed of polyethylene terepthalate with a polybutilate coating. It has been used for many years in both open and arthroscopic shoulder surgery. In the past two decades, various sutures have been introduced which claim to have higher tensile strength and superior handling characteristics to traditional sutures. Two of these sutures have become widely adopted in arthroscopic shoulder surgery. FibreWire (Arthrex, Naples, Florida, USA) is a polyethylene/polyester cobraid with a silicone coating that was introduced in 2002. Orthocord (Mitek, Raynham, Massachusetts, USA) is composed of a polyethylene fibre braided around a central polydiaxannone core with a 90% caprolactone and 10% glycolide copolymer coating [3]. In arthroscopic shoulder surgery, these sutures are commonly used in conjunction with suture anchors. The suture is free to move within the anchor eyelet, thereby subjecting the suture material to bending and frictional forces that can lead to suture wear and the formation of particulate debris [4]. Moreover, shuttling of sutures and use of a knot pusher to lay down arthroscopic knots may lead to similar frictional and abrasive forces on suture material.

Current literature suggests that suture material stimulates a foreign body reaction within synovial tissue, resulting in the recruitment of macrophages and inflammatory cells, which attempt to phagocytose the foreign material. Failure to digest the foreign body results in the formation of multinucleated giant cells which can be identified histologically. In their study in a rabbit model, Carr et al. demonstrated that eight commonly used orthopaedic sutures induce an inflammatory response in soft tissue. However, FibreWire, Ethibond, and Orthocord did not induce a significantly greater inflammatory response than other orthopaedic sutures [3]. Further studies suggest that wear debris from orthopaedic biomaterials such as ultra-high-molecular-weight polyethylene (UHMWPE)—a common component of contemporary suture materials—induces an inflammatory response, with production of inflammatory cells and inflammatory cytokines IL-1 and TNF α [5].

As part of a systematic approach to determine the causal pathways of PAGCL, the aim of this study was to determine whether suture material commonly used in arthroscopic shoulder surgery and their wear particles stimulate an inflammatory reaction in synovial tissue. Further, we sought to determine whether this inflammatory response was associated with the production of various matrix metalloproteinases (MMP) that have been implicated in cartilage destruction [6]. We utilised a murine airpouch model to examine the inflammatory response of a synovial membrane towards commonly used sutures including intact Ethibond, FiberWire, Orthocord, and FiberWire suture wear particles. We chose to study FibreWire wear particles, as Savage et al’s study demonstrated that FibreWire produces significantly more wear debris under abrasion than Ethibond and Orthocord [4]. The inflammatory response was quantified using both histological and immunohistochemical methods. We hypothesised that all intact suture material would trigger an inflammatory response and MMP production and that suture wear particles would induce a stronger biologic reaction than intact suture.

## Methods

### Study design

Fifty 12-week-old Wistar rats were randomly allocated to five groups (*n* = 10 per group). Group 1 was the control group and had no suture material implanted; group 2 had 3 cm of intact FibreWire suture implanted; group 3 and group 4 had 3 cm of intact Ethibond and Orthocord suture implanted respectively; and group 5 had FibreWire wear debris particles implanted. Particles were created by abrading 3 cm of FibreWire suture using a surgical scalpel blade, in order to create wear particles in the micron size particle range. All suture material was sterile. Study approval was obtained from the local Animal Care and Ethics committee. Experiments were performed from 2012 to 2013.

### Murine airpouches

**Fig. 1:**
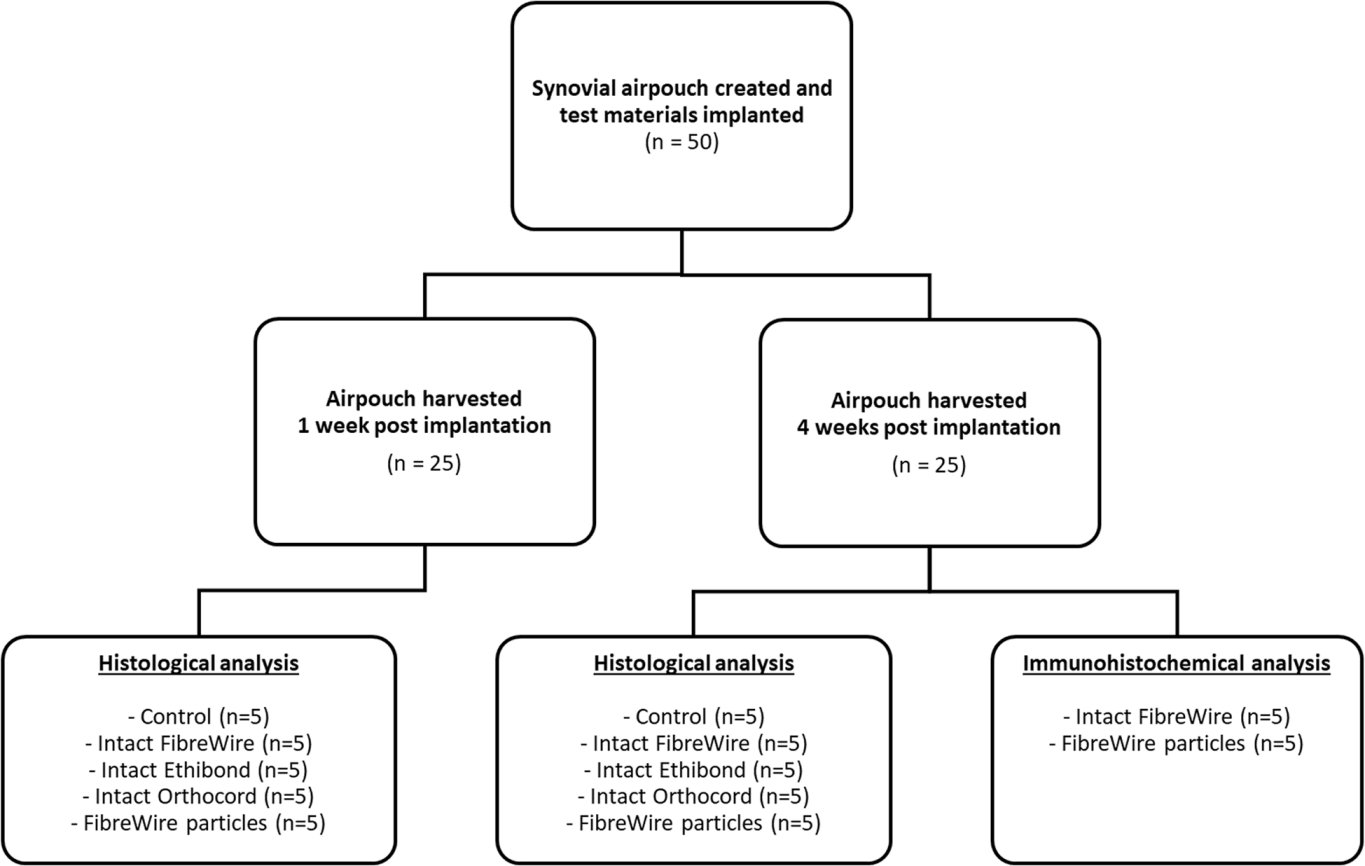
Flowchart illustrating study design

Synovial airpouches were created according to a modified protocol, as previously described by Edwards et al. [7]. This technique mechanically disrupts the subcutaneous connective tissue of the rat using subcutaneously injected air, causing accretion of macrophages and fibroblasts. This produces a cavity lined by tissue with many of the histological and electron microscopic features of synovium. The surgical procedure was performed under sterile conditions under general anaesthesia (1.5% isoflurane and 100% oxygen inhalation [2 L/min]). On day 1, an area of the dorsal skin was injected with 20 ml of air subcutaneously. The airpouches were then re-injected with 3 ml of air on day 3 to establish a definitive pouch with synovial membrane. On day 6, a 1 cm long incision was made in the airpouch and one of the test materials introduced according to groups specified. The incision was sealed using Vetbond (3 M Vetbond, 3 M Animal Care Products, MN, USA) and 5 ml of sterile phosphate-buffered saline (PBS) injected into the pouch to suspend the test materials. The control group was injected with 5 ml of sterile PBS only. Five rats from each group were sacrificed at 1 week and the remaining five at 4 weeks post-implantation (Fig. 1). All airpouches were harvested and processed for haematoxylin and eosin (H&E) histological examination. The synovial inflammatory response was determined by the number of multinucleated giant cells surrounding the suture materials. Four-week airpouch specimens from group 2 (intact FibreWire suture) and group 5 (FibreWire wear particles) underwent immunohistochemical analysis for MMP expression (MMP-1, MMP-2, MMP-3, MMP-9, and MMP-13).

### Histology

Tissue samples were fixed, dehydrated, and embedded in paraffin blocks with particular care taken to preserve the original shape of the airpouch tissue. Two separate sections were cut along the axial axis of the suture at approximately the midline of the airpouch, taking care to cut through the implanted suture material. The sections were mounted and stained with H&E. The histological sections were digitally photographed and evaluated for the presence of synovium and confirmation of implanted material and foreign body giant cell response. Multinucleated giant cells were quantified at × 20 magnification within two separate regions of interest (ROI) from each sample. The number of multinucleated giant cells was quantified by three blinded, independent observers, and the average cell count was used in the analysis.

### Immunohistochemistry

Immunohistochemistry was performed on the paraffin section for 4-week specimens from groups 2 and 5 to determine the expression of MMP-1, MMP-2, MMP-3, MMP-9, and MMP-13. This was performed using a previously described technique [8, 9]. Briefly, slides were first de-paraffinised and then treated with a citrate-based (neutral pH) antigen retrieval solution (DAKO Pty Ltd., Glostrup, Denmark) at 95 °C for 20 min. Upon cooling to room temperature, endogenous peroxidase was quenched by 0.3% hydrogen peroxide in 50% methanol for 10 min. Slides were rinsed in distilled water and washed in PBS with 0.2% Tween-20 (PBS-T). Primary mouse monoclonal antibodies against MMP-1 (IM35L), MMP-2 (IM33L), MMP-3 (IM36L), MMP-9 (IM60L), and MMP-13 (IM44L) (Oncogene Research Products, Boston, MA, USA) and nonimmunized mouse immunoglobulin (IgG) (DakoCytomation, Glostrup, Denmark) against negative controls were applied to all the sections and left overnight at 4 °C in humidity chambers. The concentrations of the primary antibodies were 0.1 μg/ml for MMP-1, 0.2 μg/ml for MMP-2, MMP-3, and MMP-13, and 0.5 μg/ml for MMP-9. The final concentration for IgG was 0.1 μg/ml. The following day, the slides were washed three times in PBS-T and the DakoCytomation Envision^+^ System-HPR Labelled Polymer specific for mouse (K4001) (DakoCytomation, Glostrup, Denmark) was applied at room temperature for 1 h. Sections were washed in PBS-T four times prior to the application of a substrate-chromogen system, DAKO® Liquid diaminobenzidine (DAB) (K3468, DakoCytomation, Glostrup, Denmark). After 30 min, the reaction was terminated by immersing the slides in PBS. The sections were counterstained with Harris Haematoxylin. MMP expression was quantified using software written in Matlab 7.0 (The MathWorks, Natick, MA, USA), designed to select ROIs based on colour matching from digitally captured images. The software processes images of interest and converts the colour space from red/green/blue (RGB) to Commission Internationale d’Eclairage L*a*b (CIELAB) [10]. One ROI (original magnification × 20), most representative of the entire section, was assessed for each specimen (*n* = 5 per group). The amount of stained area within the giant cells and immediately surrounding, as determined by histomorphometry, was compared to the area covered by the suture material to yield the ratio of MMP stained to suture material stained. This resultant ratio was considered representative of MMP activity.

### Statistical analysis

Quantitative data was analysed using analysis of variance with post hoc pairwise comparisons to determine any statistical differences between groups. Differences were considered significant at *p* ≤ 0.05. Statistical analysis was performed using SPSS version 18.0 for Windows (SPSS Inc., Chicago, Illinois, USA).

## Results

### Histology

**Fig. 2:**
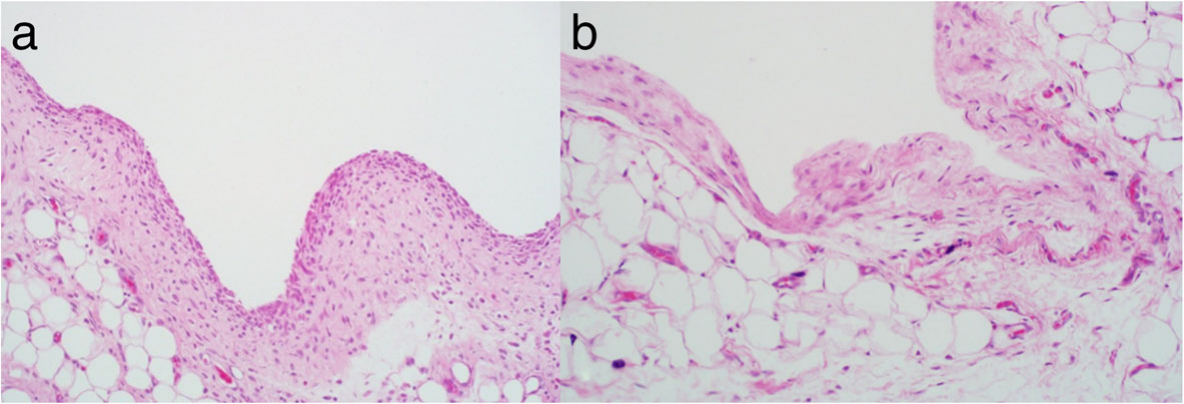
Histology images of synovial lining in the control group (H&E staining, × 20 magnification). **a** 1 week. **b** 4 weeks. Synovial lining is characterised by fibrous tissue populated with fibroblast-like cells and histiocytes. The lining was thicker and more densely populated with cells at 1 week than 4 weeks

Implantation of suture materials into the airpouch did not cause clinical signs of infection in any specimen. Synovial environment was confirmed by the presence of a synovial lining on the inside of the capsule created within the airpouch. In the control group, 1 week post-implantation, the lining membrane was characterised by fibrous tissue highly populated with fibroblast-like cells and histiocytes (Fig. 2a). At 4 weeks post-implantation, the lining was still present however cellular density and membrane thickness were decreased (Fig. 2b).

**Fig. 3:**
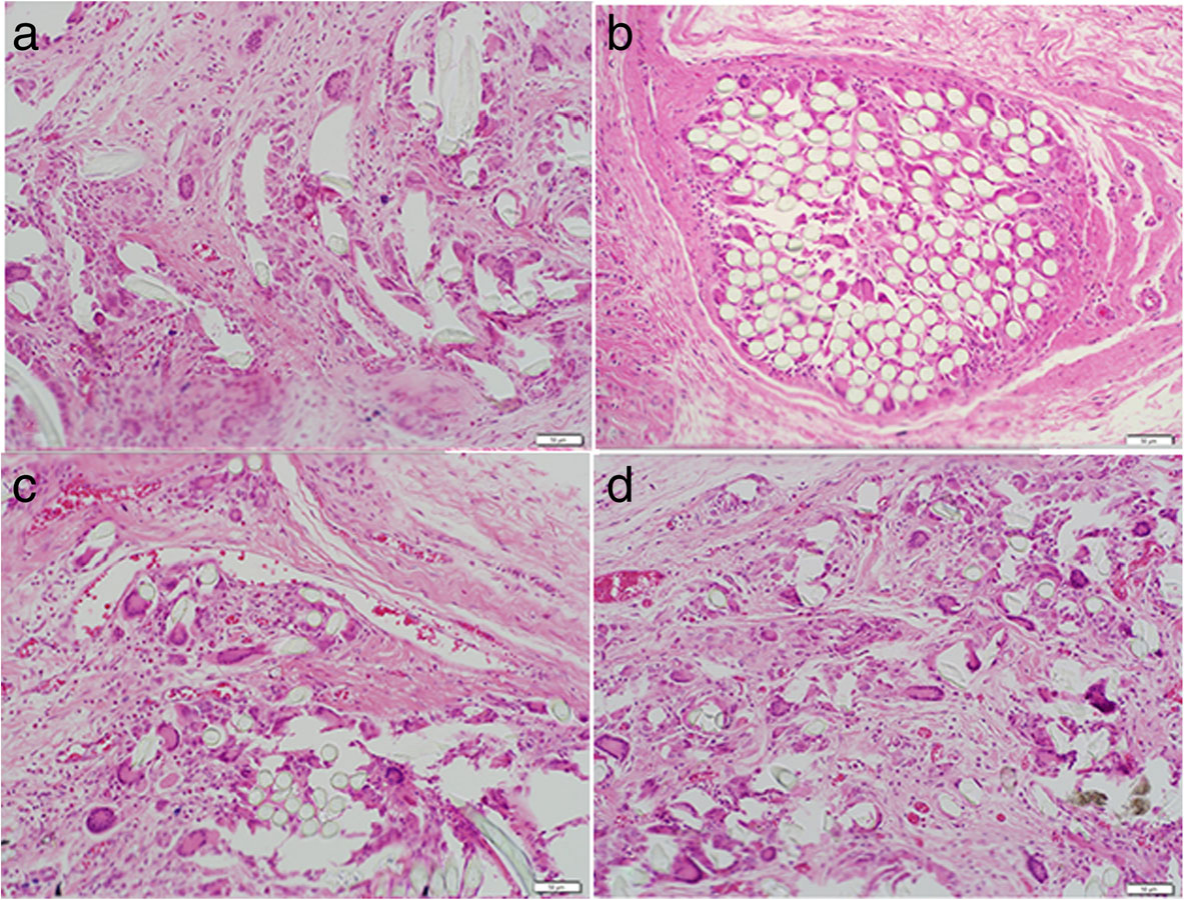
Histology images in test groups at 4 weeks post-implantation (H&E staining, × 20 magnification). **a** Orthocord intact. **b** Ethibond intact. **c** FibreWire intact. **d** FibreWire particles. Multinucleated giant cells can be seen at the suture-soft tissue interface in all groups

**Fig. 4:**
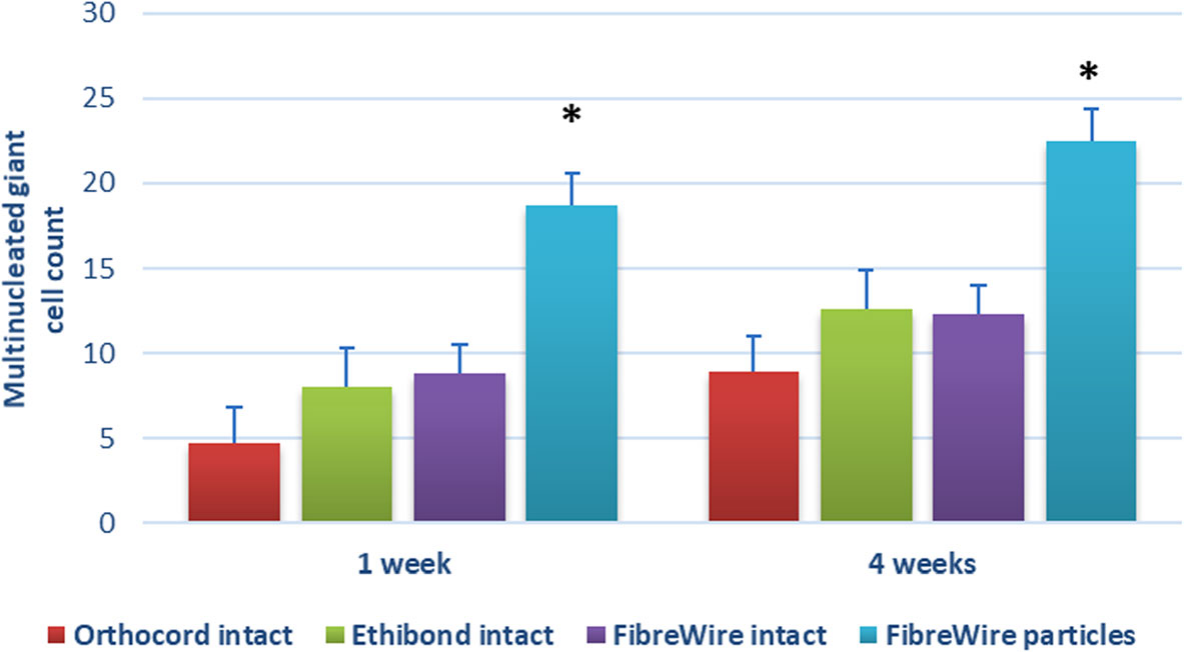
Number of multinucleated giant cells for all test groups at 1 and 4 weeks post-implantation. A statistically significant difference was found between FiberWire intact suture and FiberWire particles at both time points. There were no significant differences found in the number of multinucleated giant cells counted between intact suture groups at either time point. All data are expressed as mean ± standard error. *p < 0.05

Multinucleated giant cells were not observed in any sections of the control specimens. A foreign body reaction, including multinucleated giant cells, was seen in all groups where test material was implanted, indicating a greater inflammatory response compared to controls (Fig. 3). Of the test groups, Orthocord suture elicited the least number of giant cells at both time points (1 week: 4.70 ± 1.93; 4 weeks: 8.93 ± 1.01). The mean number of giant cells was slightly higher for both Ethibond (1 week: 7.97 ± 2.61; 4 weeks: 12.57 ± 1.30) and FiberWire (1 week: 8.77 ± 2.37; 4 weeks: 12.27 ± 2.80). These differences were not statistically significant. Significantly more giant cells were observed in FiberWire wear particles group (group 5) compared with FiberWire intact suture group (group 2) at week 1 (18.73 ± 2.29; *p* = 0.021) and week 4 (22.50 ± 1.01; *p* = 0.003) (Fig. 4).

### Immunohistochemistry

**Fig. 5:**
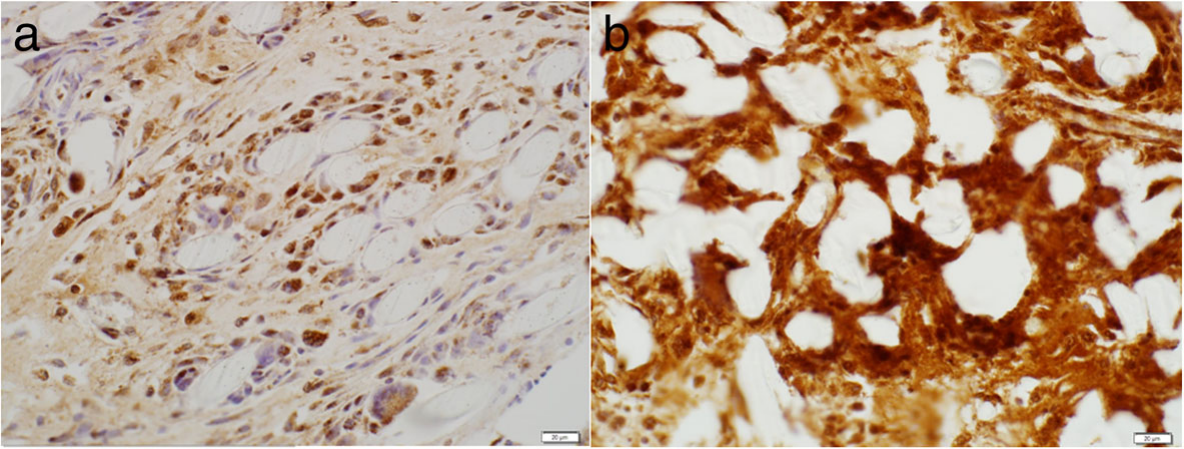
Immunohistochemistry slides showing expression of MMP-1 in response to **a** intact FibreWire and **b** FibreWire particles

**Fig. 6:**
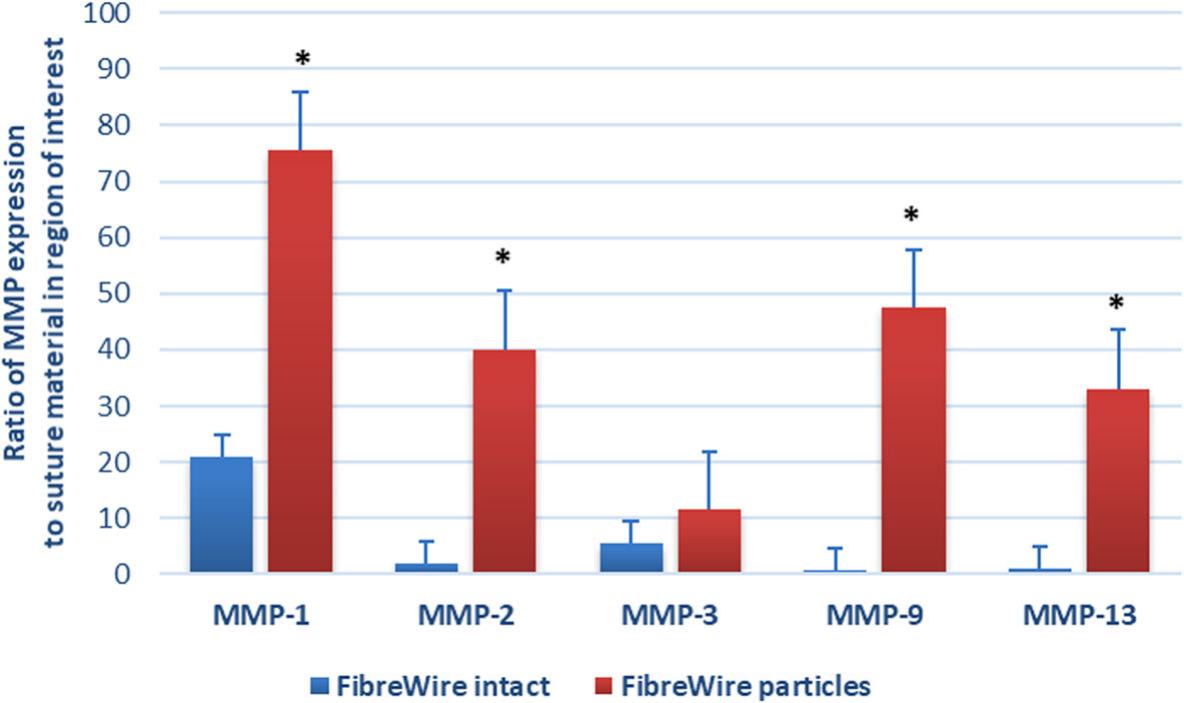
Quantitative analysis of MMP expression at 4 weeks post-implantation. A statistically significant difference was recorded at 4 weeks between FiberWire intact and FiberWire particles for MMP-1 (*p* = 0.024), MMP-2 (*p* = 0.009), MMP-9 (*p* = 0.002), and MMP-13 (*p* = 0.007). The difference for MMP-3 was not significant (*p* = 0.058). All data are expressed as mean ± standard error. *p < 0.05

Nonimmunized mouse immunoglobulin was used as a negative control to confirm that the primary antibody was specific. No staining was observed in the negative controls. Quantitative analysis of immunohistochemical staining for MMP-1, MMP-2, MMP-9, and MMP-13 expression demonstrated a significantly higher ratio of MMP stained to suture material stained in the FiberWire particles group (75.53 ± 16.04, 40.09 ± 8.05, 47.49 ± 6.35, 33.15 ± 6.35 for MMP-1, MMP-2, MMP-9, and MMP-13, respectively) compared to the intact FiberWire group (21.05 ± 5.94, 1.87 ± 0.68, 0.64 ± 0.31, 0.95 ± 0.27 for MMP-1, MMP-2, MMP-9, and MMP-13, respectively) at 4 weeks (*p* = 0.024, *p* = 0.009, *p* = 0.002, and *p* = 0.007 for MMP-1, MMP-2, MMP-9, and MMP-13, respectively) (Fig. 5). No statistical difference was noted between the groups for MMP-3 expression (5.65 ± 1.93 and 11.42 ± 1.74 for FiberWire intact and FiberWire particles group respectively; *p* = 0.058) (Fig. 6).

## Discussion

Despite it being a significant cause of morbidity following arthroscopic shoulder surgery, little is known about the aetiology and molecular pathways underlying PAGCL. Hedbom and Hauselmann suggest that chondrolysis is promoted by molecular and biochemical signals received from the inflamed synovial membrane [11]. Subsequently, an inflammatory response triggered by suture material or its wear debris may play a potential role in the development of PAGCL.

In this study, we examined the biologic response of synovial tissue to commonly used orthopaedic sutures material. A limitation of this study is that only three types of sutures were tested and only Fibrewire was examined in the particle form. These three sutures were chosen as they were felt to reflect the most commonly used sutures in current shoulder surgical practice. However, it is important to note that a similar inflammatory response may be seen with other suture materials not tested in our study. As has been highlighted above, FibreWire wear particles were chosen in light of previous studies demonstrating increased abrasive wear compared with Ethibond and Orthocord [4]. Ideally, future studies should examine the bioreactivity of wear particles for all sutures being investigated, as sutures with a different material composition may elicit a different host response in particle form.

This study confirmed that Ethibond, FibreWire, and Orthocord sutures stimulate an inflammatory reaction in synovial tissue, as demonstrated by the presence of multinucleated giant cells. In addition, this study demonstrated a significantly increased inflammatory reaction to FibreWire wear particles compared to intact FibreWire. Moreover, the significant increase in MMP production in response to FibreWire wear particles compared to intact FibreWire demonstrated in this study points to a potential aetiological pathway in the development of chondrolysis.

The findings of the current study are consistent with those of Carr et al., who also demonstrated an increased number of multinucleated giant cells in response to eight commonly used orthopaedic suture materials implanted into the dorsal fascia of a rabbit [3]. However, Carr et al.’s study tested only intact suture material rather than suture wear debris. In addition, the tests were conducted in the dorsal fascia of the rabbit, which does not recreate the synovial tissue that is produced by using a murine airpouch model. Accordingly, the results of the present study may more accurately reflect the biologic response to suture material specifically in synovial spaces such as joints and bursae.

The augmented inflammatory response to wear particles from polymers commonly used in orthopaedic surgery has been previously demonstrated in a murine airpouch by Wooley et al. [5]. This study found that particles of UHMWPE, polymethylmethacrylate, cobalt-chrome, and titanium alloy elicited a significant increase in the number of inflammatory cells and inflammatory cytokines (IL-1, TNFα) when implanted into a murine airpouch. The present study confirms that wear particles from FibreWire suture produce histological evidence of an inflammatory reaction. Moreover, this inflammatory response is significantly greater than that produced in response to intact FibreWire suture, as demonstrated by a statistically significant increase in the number of multinucleated giant cells. To our knowledge, the present study is the first to demonstrate this finding.

In this study, we examined the activity of several MMP subtypes: MMP-1 (collagenase 1), MMP-2 (gelatinase A), MMP-3 (stromelysin-1), MMP-9 (gelatinase B), and MMP-13 (collagenase 3). Evidence suggests several of these MMP subtypes can mediate the destruction of articular cartilage by initiating proteolysis of the extracellular matrix proteins that regulate the biomechanical responses of articular cartilage [6, 12, 13]. Each MMP subtype has been associated with a particular role in chondrolysis. MMP-1 is significantly increased in chondrolysis and has been shown to break down cartilage extra-cellular matrix by cleaving type II collagen. MMP-2 and MMP-9 degrade denatured type II collagen that was initially cleaved by activated MMP-1 [6]. They also degrade aggrecan, a large proteoglycan critical for cartilage structure and mechanical properties. MMP-3 has been shown to degrade cartilage proteoglycans as well as the link protein which stabilises the non-covalent interactions between aggrecan and hyaluronan. MMP-13 can degrade both type II collagen and aggrecan. The present study used immunohistochemical methods to quantify the activity of these matrix metalloproteinase subtypes in the intact FibreWire and FibreWire particles groups at 4 weeks post-implantation. There was significantly greater production of MMP-1, MMP-2, MMP-9, and MMP-13 in the FibreWire particles group compared to the intact FibreWire group. A twofold increase was also noted for MMP-3 in the particles group. Although the *p* value of 0.058 approached significance, it is possible that a larger sample size may have been required to detect a statistically significant difference. Our findings support the notion that several MMP subtypes are produced as a result of the increased immune response to FibreWire wear particles. We hypothesise that this is likely due to the fact that the smaller particles associated with wear debris offer more surface area for macrophage attachment and initiation of the inflammatory response. Previous studies have demonstrated that suture material can increase MMP production in tendon [14]. However, as far as we are aware, this is the first study to demonstrate an increase in MMP expression in synovial tissue exposed to both intact suture and wear particles. This finding gives insight into a possible molecular mechanism by which suture material may contribute to the development of chondrolysis.

In our study, we did not attempt to quantify the expression of proinflammatory cytokines such as interleukin-1β (IL-1β), IL-6, and tumour necrosis factor α (TNFα). A previous study by Lock et al. demonstrated that FibreWire significantly increased expression of TNFα in vitro compared to controls, but not other pro-inflammatory cytokines including IL-1α, IL-1β, or IL-8 [15]. There is a substantial amount of experimental and clinical evidence that these pro-inflammatory cytokines are crucial in mediating inflammation and cartilage destruction in human osteoarthritis by upregulating the gene expression and synthesis of MMP subtypes [16–18]. It is probable that this cascade of events is involved in PAGCL.

Our study has a number of limitations. The use of a murine subcutaneous airpouch to investigate the bioreactivity of different surgical materials in synovial tissue is a well-established, valid model [19, 20]. However, it is unknown whether the results are completely translatable to humans, and this may therefore limit the study’s application to the clinical situation of PAGCL. Secondly, we studied the inflammatory response at two time points. The first time point of 1 week was chosen to investigate the initial reaction to foreign material, while the 4-week time point was considered adequate to mimic a more chronic setting. In the clinical setting, however, most patients who develop PAGCL will present with symptoms approximately 3 months following arthroscopy [2]. Therefore, future studies would benefit from investigating the inflammatory response for a more prolonged period. Finally, as discussed above, future studies should examine the bioreactivity of all sutures commonly used in arthroscopic shoulder surgery and their wear particles.

These findings have significant clinical implications for orthopaedic surgeons. Firstly, this study highlights the importance of avoiding damage to suture material during arthroscopic procedures. Surgeons should be vigilant about monitoring for frayed or excess suture material intraoperatively, use sharp instruments when cutting sutures, and use a knot pusher judiciously due to its propensity to cause suture abrasion. Moreover, these findings have implications for surgical technique and suture anchor design, with Bardana et al’s study demonstrating that suture abrasion is significantly reduced when sutures are manipulated in line with the anchor’s eyelet, with minimal rotation or angulation [21]. In addition, concerns over suture wear debris may make the use of knotless suture anchor systems preferable, by negating the movement of suture material through an eyelet and avoiding knot tying with a knot pusher, thereby minimising abrasion.

## Conclusions

In summary, PAGCL is a significant complication seen in arthroscopic shoulder surgery, whose aetiology is poorly understood and likely multifactorial. Orthopaedic biomaterials, especially high strength suture material, may play a role in the development of PAGCL. Our study demonstrates that sutures commonly used in arthroscopic shoulder surgery, and their wear particles, elicit an inflammatory response and expression of chondrolytic MMPs in synovial tissue. This increase in MMP production may be a plausible molecular mechanism by which suture material may contribute to the pathogenesis of PAGCL. Further studies are required to more completely elucidate the factors contributing towards this devastating complication of arthroscopic shoulder surgery.

## Abbreviations

H&E: Haematoxylin and eosin
IL: Interleukin
MMP: Matrix metalloproteinase
PAGCL: Post-arthroscopic glenohumeral chondrolysis
PBS: Phosphate-buffered saline
ROI: Region of interest
TNF: Tumour necrosis factor
UHMWPE: Ultra high molecular weight polyethylene
